# Factors that Affect Outcome of Ultrasound-Guided Radiofrequency Ablation of Renal Masses

**DOI:** 10.3390/curroncol31090392

**Published:** 2024-09-10

**Authors:** Galyna Zinko, Marianna Hrebenyuk, Anders Kjellman, Yngve Forslin, Martin Delle

**Affiliations:** 1 Department of Radiology, Karolinska University Hospital, 141 86 Stockholm, Sweden; galyna.zinko@regionstockholm.se (G.Z.); yngve.forslin@regionstockholm.se (Y.F.); 2 CLINTEC (The Department of Clinical Science, Intervention and Technology), Karolinska University, 141 86 Stockholm, Sweden; marianna.hrebenyuk@regionstockholm.se (M.H.); anders.kjellman@regionstockholm.se (A.K.); 3 Department of Urology, Karolinska University Hospital, 141 86 Stockholm, Sweden

**Keywords:** interventional oncology, ablation procedures, renal cancer, tumor, percutaneous, ultrasound

## Abstract

The purpose of this study was to examine the factors influencing the efficacy and safety of the ultrasound-guided radiofrequency ablation of renal tumors. Between January 2010 and December 2018, 159 patients with renal tumors treated with ultrasound-guided percutaneous radiofrequency ablation at our institution were included in this study. Biopsies were performed for histopathological analysis prior to each ablation. Patients underwent computed tomography follow-ups at 3, 6, and 12 months and were subsequently observed on an annual basis. The primary efficacy rate (i.e., residual tumor), local tumor progression, morbidity and mortality, and possible outcome predictors (age, body mass index, gender, tumor size, tumor location, tumor characteristics, ablation temperature, and reported technical problems) were analyzed using binary logistic regression. At the first follow-up, 3 months after ablation, the primary efficacy rate was 79%. Two percent of the tumors showed local tumor progression during the whole follow-up. Tumor proximity to the collecting system and the final temperature in the ablation region were associated with the occurrence of residual tumor (OR = 2.85, *p* = 0.019 and OR = 4.23, *p* = 0.006, respectively). A similar trend was shown for tumors larger than 3 cm (*p* = 0.066). A short distance to the collecting system and the ablation temperature were significantly related to the occurrence of residual tumors after the radiofrequency ablation of small renal masses. The ultrasound guidance used in our study has a lower primary efficacy rate than the computed tomography guidance used in comparable studies.

## 1. Introduction

The increasing rate of cross-sectional imaging examinations performed has contributed to the increased detection of small renal masses [1,2]. These incidental findings often create a challenge regarding their treatment strategy. For T1a lesions, partial nephrectomy is regarded as the gold standard [3]. However, in elderly patients and/or patients with comorbidities, active surveillance may prove a viable alternative for tumors less than 4 cm in size, as even though about 70% are malignant, they seldom give rise to metastases [1,4]. Thus, the risk for morbidity and mortality from an untreated renal mass during active surveillance must be compared to the risk associated with surgical intervention [5].

Thermal ablation (TA) is a less invasive treatment option compared to partial nephrectomy. Over the last decade, several studies have shown comparable outcomes for T1a renal cell carcinoma (RCC) in terms of effectiveness and renal function, as well as significantly lower complication rates [5,6,7]. These patients also require shorter periods of hospitalization [5,7,8,9]. Focal ablative therapies have also been shown to have long-term oncological values relative to that of partial nephrectomy for small renal masses [7,10,11]. It has, however, been argued that the results from these retrospective comparative studies are not reliable due to the high risk of bias [12].

Percutaneous radiofrequency ablation (RFA) can be performed by means of ultrasound (US) guidance and/or computer tomography (CT) guidance. The key issue is the need to achieve an adequate needle position to perform a complete ablation. The tumor’s size and location within the kidney and patient habitus are some of the factors that may potentially influence needle positioning accuracy and the outcome of the procedure [13,14].

The main outcome of this retrospective study was the effectiveness of US-guided RFA in terms of its primary efficacy, local tumor progression, and complications. How the tumor’s size and location within the kidney, as well as the technical problems encountered during the US-guided ablation, affected the outcome was also analyzed.

## 2. Materials and Methods

### 2.1. Patient Selection and Tumor Characteristics

Between June 2010 and December 2018, a total of 183 patients subjected to the RFA of their renal tumors at our institution were retrospectively reviewed. The indication for RFA included a tumor size < 4 cm that (CT-defined) was deemed accessible for percutaneous ablation, patients not suitable for surgery due to comorbidities and frailty (ASA-score), impaired renal function, and, in some cases, patients’ preferences. The criteria were in line with the recommendations from the European Association of Urology [3]. Larger tumor sizes were accepted for ablation only when surgery was too high of a risk. The treatment decision was made in consensus between interventional radiologists and urologists at a multidisciplinary conference. The final decision on the choice of treatment was made after clinical evaluation and discussion with the patient. Fragile patients with slow-growing tumors who were indicated for active surveillance were not included in the study.

**Figure 1:**
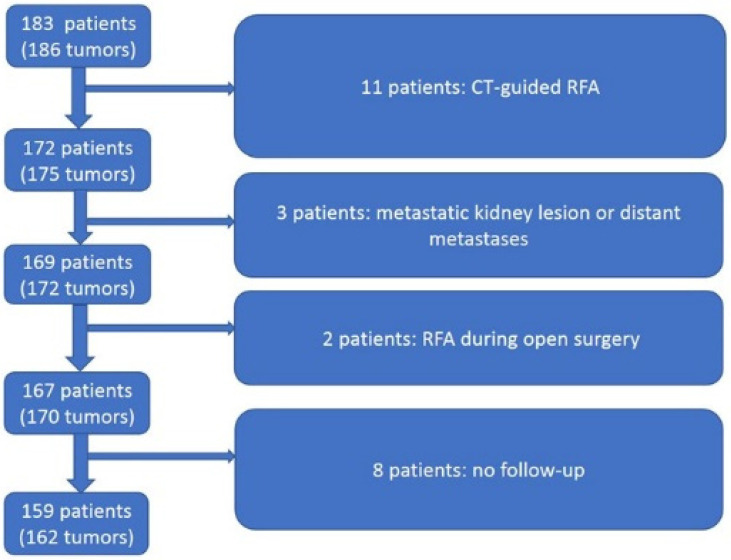
Description of patient exclusion.

This study was approved by our local Ethics Review Board. Patients treated for a metastatic lesion in their kidney from another malignancy, patients with distant metastases, or patients who had an RFA performed during open surgery were excluded from this analysis. If the visualization with US was insufficient as a guiding tool, or the anatomy difficult to delineate, CT imaging was used for guidance instead and the patient was excluded from the analysis. Moreover, patients were excluded in cases of inadequate follow-up (patients moving to other regions) or if a reliable follow-up had not been possible to perform (with CT, magnetic resonance imaging (MRI), or US with a contrast agent). In total, 24 patients were excluded from analysis (Figure 1). One hundred and fifty-nine patients were thus included in the study.

The demographic information of the study population is detailed in Table 1. Prior to RFA, all patients underwent examination with contrast-enhanced CT (multiphase). Their tumors were characterized as shown in Table 2.

### 2.2. RFA—Technical Aspects

A US-guided 18G core needle biopsy was performed 2–3 weeks prior to the RFA procedure. If the histopathological analysis was inconclusive, another biopsy was taken in the same session as the RFA procedure, according to the clinical routine of our institution.

All patients were treated under general anesthesia. Ablations were performed by means of a Cool-tip™ RF Ablation System E Series, Covidien/Medtronic (Kista, Sweden).

**Figure 2:**
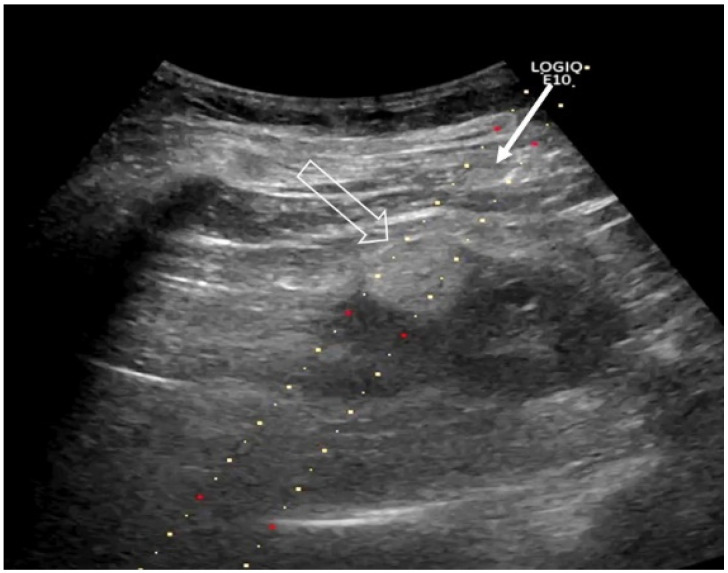
Technique of US-guided RFA. This unenhanced US prior to RFA shows a 1.5 cm renal cell carcinoma (big arrow). The long arrow shows the needle guidance given for the electrode positioning.

US guidance (Acuson Sequoia Ultrasound System, Siemens Healthcare Solna, Sweden, and GE Logiq 9 and Logiq 10, GE Healthcare, Danderyd, Sweden) was used to place the RFA electrode (17 Gauge) centrally in the tumor (Figure 2).

In a few patients, contrast-enhanced US (CEUS) was used to optimize tumor delineation. The number of ablations performed in one procedure was based on the size of the tumor and whether a sufficient temperature level was achieved. For tumors larger than 3 cm, 2–3 overlapping ablations were performed, with 12 min per ablation. Alternatively, a cluster electrode (consisting of three connected electrodes) was used, which had an ablation time of 16 min. Hydrodissection was performed when the ablation zone endangered adjacent organs. The ablation temperature was directly measured via the RF electrode and recorded at the end of the ablation. A temperature of 65 °C was set as the limit for an adequate ablation (manufacturer’s recommendations) [15]. To avoid the potential local spread of tumor cells, tract ablation was performed as the electrode was withdrawn. Finally, local anesthesia (Lidocaine 10 mg/mL Aspen Nordic, Ballerup, Denmark) was injected at the puncture site.

Technical difficulties, described by the operators in the procedure reports, were recorded to analyze how technical/practical issues related to the US-guided ablation could affect the primary outcome in terms of efficacy. Complications were described according to the Modified Clavian system [16,17]. Thirty-day mortality and morbidity data were collected, as well as long-term survival rates.

### 2.3. Follow-Up

CT with a multiphasic contrast protocol (including non-enhanced, corticomedullary, nephrographic, and excretory phases) after the RFA was used as the follow-up modality. Exams were performed at 3, 6, and 12 months after the ablation procedure and annually thereafter for up to 5 years. For the few patients with an iodine contrast allergy or with a glomerular filtration rate lower than 45 mL/min, MRI or contrast-enhanced US was used.

The primary efficacy rate was defined as the percentage of completely ablated lesions at the first follow-up (i.e., three months after the RFA session). The secondary efficacy rate was the percentage of cases with fully ablated residual tumors after all RFA procedures. Local tumor progression was used to denote the reappearance of tumor in the ablation region after at least one contrast-enhanced follow-up CT had documented adequate ablation and an absence of viable tissue in the target tumor [18].

**Figure 3:**
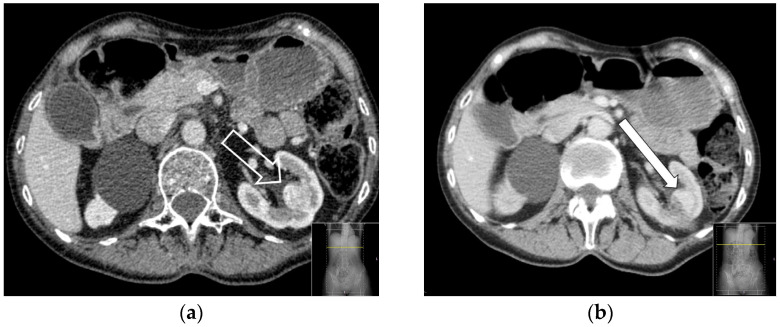
(**a**,**b**) Example of the transverse section of a contrast-enhanced CT of a patient before and after the RFA. (**a**) Before the RFA there is an enhanced RCC (big arrow) in the left kidney. (**b**) Three months later, the follow-up CT shows the remaining enhancement (long arrow) in the lateral aspect of the treated tumor (residual tumor).

The primary efficacy and local tumor progression were defined according to the criteria of the Cardiovascular and Interventional Radiological Society of Europe (CIRSE) guidelines [19]. The finding of a contrast-enhanced lesion (>15 HU compared to a non-enhanced phase) in an initial follow-up exam was regarded as a residual tumor (Figure 3a,b). If a contrast-enhanced lesion was detected in subsequent controls, it was recorded as local tumor progression, as described above.

### 2.4. Data Analysis and Statistics

The statistical analysis was performed according to a predefined analysis plan. Factors influencing the risk of residual tumor were analyzed by multivariate logistic regression. BMI, age, sex, exophytic/endophytic tumor, localization of the tumor, proximity to calyces, size, final ablation temperature, and technical difficulties were analyzed. A multivariate logistic regression was also performed in a subgroup analysis of the tumors with Fuhrman grading.

By stepwise backward selection, the four most important variables were kept in the model. The fit of the model was analyzed by ROC. Analyses were carried out with STATA v16 software (Stata Corp, LP Lakeway, TX, USA) and SPSS version 28.0 for Mac (IBM Corp., Armonk, NY, USA). The odds ratios (ORs), with 95% confidence intervals and *p*-values, are presented. The results were considered statistically significant if the *p*-value was lower than 0.05. This statistical analysis used the results from the first ablation session for the determination of the factors that influenced the primary effectiveness rate.

## 3. Results

A total of 183 patients (122 men, 61 women) underwent RFA during the study period. Twenty-four patients were excluded from the analysis, as described in Figure 1. Therefore, 159 patients (105 men, 54 women), with a total of 162 lesions treated with RFA, were included in our analysis. The mean age (range) of these patients was 73 (35–89) years. The mean tumor size (range) was 24 cm (1.0 cm–5.5 cm).

### 3.1. Renal Tumors’ Histology

A core needle biopsy was taken from 160 of 162 lesions, with representative samples seen in 127 lesions (80%), which includes a second biopsy attempt during the RFA procedure if needed. To not extend the time to treatment, the clinical routine was to perform the RFA directly after a second attempt to obtain a representative biopsy. Details of the histopathological analysis are summarized in Table 3. The Fuhrman grade was described in 56 histological reports (49% of representative biopsies). Grade 1 and grade 2 tumors were the most common (24 and 27 samples, respectively), while grade 3 was described in only 3 samples.

### 3.2. Ablation Temperature

The ablation temperature was reported in 151 of 162 ablations. The final temperature was 65 °C or more in 132 (82%) and less than 65 °C in 19 (11%) of the procedures. For details see Table 4.

### 3.3. Technical Difficulties during the Procedure

Technical difficulties were issues reported during the US-guided ablation that were considered able to potentially affect the outcome, such as additional ablation due to the suspicion of remaining tumor, problems with positioning the antenna (the mobility of the kidney), problems in achieving an adequate visualization of the tumor, or a prolonged procedure due to a low ablation temperature. One procedure was interrupted before completion (due to patient movement resulting in electrode dislocation). Technical difficulties were described in 51 of the 162 (32%) procedures, as shown in detail in Table 5.

Hydrodissection was performed in two cases where the colon was near the ablation area.

### 3.4. Complications

A total of 26 (16%) cases with complications were reported, out of which 23 (14%) had early complications (within 30 days). Only 5% of all patients had complications that required additional measures or prolonged hospital stays. Most cases (10%) were classified as Clavien 1 (mostly problems related to urinary catheterization and local skin reactions at the puncture site).

Six patients had Clavien 2 complications: a local inflammatory reaction of the colon that was treated with antibiotics (two cases), pyelonephritis and fever treated with antibiotics (two cases), or perirenal hematoma within the Gerotas fascia with following blood transfusion (two cases). One patient had a Clavien 4 complication, a problem with extubation, and was referred to the ICU for a short period the same day. The early complications are summarized in Table 6.

Three patients developed ureteral stricture as a late complication. One of the patients had pronounced parenchymal reduction and underwent a nephrectomy due to this (Clavien 3). The other two had no symptoms and no additional measures were required.

### 3.5. Efficacy and Local Tumor Progression

The median follow-up was 20 months (with a total span of 30–101 months).

The 30-day mortality rate was 0%. Thirteen (8%) patients died during the follow-up period. None of the patients suffered RCC-related mortality.

One patient was diagnosed with metastases during their follow-up. The patient had an RCC of 2 cm with a histological finding of clear cell carcinoma Fuhrman grade 2. A residual tumor was observed on their initial follow-up CT scan and open resection was performed 7 months after the RFA. Lung metastases (a histologic finding of clear cell carcinoma) were detected on a CT scan 5 months after the surgery.

During follow-up, the primary efficacy rate was 79, i.e., 128 of 162 lesions had no signs of residual tumors at first follow-up. Eleven patients were treated with the cluster electrode technique due to a tumor size ≥4 cm. Of these, five patients (45%) had a residual tumor at their first CT follow-up. Four ablated lesions (2%) showed local tumor progression, i.e., a reappearance of tumor growth after a first tumor-free CT control at 3 months.

Residual tumors and local tumor progression were treated either with additional ablation, surgery, or active surveillance, as demonstrated in Table 7. Nineteen patients with residual tumors were treated with an additional ablation. Of these, five patients were still diagnosed with residual tumors at a following CT control and were successfully treated with a third ablation (three patients) or open surgery (two patients). After all additional ablations of the residual lesions, secondary efficacy was 89% (145 lesions of 162 had no signs of remaining contrast enhancement).

### 3.6. Factors Influencing the Outcome

The analysis of the factors that might influence the outcome of the ablation was made by logistic regression. The results are shown in Table 8. The ROC for the model that kept the four most influential variables for residual tumors provided an AUC of 0.770.

As follows, patients with a final ablation temperature below 65 °C had four times greater odds that the procedure would result in an incomplete ablation (*p* = 0.006; OR = 4.23). Proximity to the collection system (less 4 mm) was another factor that significantly correlated with an increased risk of residual tumors (*p* = 0.019; OR = 2.85). As only 49% of the histological reports had a Fuhrman grading (56 patients), a subgroup analysis was performed separately, which did not show a significant association of this grading with the occurrence of residual tumors (*p* = 0.075; OR = 2.56).

As shown in Table 8, there was a non-significant trend in the correlation between tumor size (>30 mm) and primary efficacy, i.e., residual tumors after the procedure (*p* = 0.066). This was not found in the relationship between the reported technical difficulties and residual tumors (*p* = 0.177) However, in 10 cases of these residual tumors, the ablation zone did not include the lesion. In four of these cases, technical difficulties were reported, three cases experienced difficulties in visualizing the tumor, and one case included a dislocation of the kidney while positioning the antenna.

There was no significant correlation between incomplete ablation and any of the other evaluated factors (sex, age, BMI, exophytic/endophytic tumors, localization of tumors, right/left side of the lesion).

Supplementary Table S1 shows an overview of the patient and tumor characteristics of the following two groups: complete and incomplete ablation (including local tumor progression).

## 4. Discussion

This single-center, retrospective study shows a somewhat higher incidence of residual tumors in patients treated with US-guided RF ablation compared to several previous studies [6,20,21,22,23], but similar results to a recent study from Poland [24]. There are several factors that might contribute to this finding; factors that seem related to the US technique as well as the tumor’s size and location. However, the local tumor progression rate and procedure-related complications were both observed to be low, with the latter highlighting the minimally invasive nature of this treatment option.

Several factors have the potential to influence the outcome after thermal ablation. The accurate positioning of the electrode centrally in the lesion is crucial for complete tumor ablation [13]. There is currently no general agreement concerning the ablative margin when treating RCC with RFA. However, an ablation margin of at least 0.5 cm around the tumors has been considered adequate during RFA in the liver, although some renal tumors have been suggested to need less of a margin [25]. In addition, the inappropriate positioning of the antenna may damage nearby structures (the colon, proximal ureter, renal pelvis, adrenals, and pancreas).

Therefore, accurate imaging during the procedure is essential to delineate the anatomy and localize the electrode’s position. In this respect, CT and/or US as guiding tools have been utilized and evaluated in the literature. US has the advantage of offering a live image while using needle guidance. The Doppler and US contrast techniques visualize vessels in detail. However, small endophytic lesions might be difficult to delineate. Additionally, in obese patients, if the lesion is covered by gas-filled bowel, the image quality may be impaired and the precision of the placement of the RFA electrode may be jeopardized. No randomized studies comparing CT and US guidance are available, to our knowledge, and the technique used depends on local routines and competence.

Interestingly, the advantages of US as a tool used to delineate the anatomy and extension of renal tumors during laparoscopic partial nephrectomies have recently been reported [26]. In this study, the use of intraoperative US contributed to lower blood loss, shortened operative durations, the optimization of surgical margins, and enhanced procedural safety and precision. Intraoperative US enables a higher resolution due to the reduced distance between the probe and the target organ relative to the longer distance between the probe and target organ during percutaneous intervention, which may possibly be affected by shadowing artifacts from covering ribs or organs. This is reflected in the present study by an efficacy of 79%.

Although our study did not directly show any significant correlation between most of the evaluated factors and incomplete ablation, the relatively high incidence of residual tumors still suggests that the shortcomings of the US technique may worsen the outcome of the RFA treatment. The primary efficacy rate was 79%, i.e., the 3-month CT follow-up showed insufficient ablation in 21% of cases, which was diagnosed as residual tumor growth. This is a somewhat higher number of incomplete ablations than that found in other studies where CT guidance was used for electrode positioning [13,27,28]. Acosta Ruiz et al. [13] reported 83% primary efficacy after CT-guided RFA, which is somewhat higher than our findings, while Zhou et al. [27] presented a primary efficacy of 95%. Similar results are reported by McClure et al. [28] and Marshall et al. [29], where a combination of US and CT guidance resulted in a primary efficacy of 86% and 90%, respectively. However, a previous study with US as the only imaging modality for the guidance of RFA in 32 patients showed a primary efficacy rate of 86% [23], although the study included only a scarce number of patients. A more recent study with 191 tumors that were ablated with only US guidance showed a primary efficacy of 73%, which was somewhat lower than our results [24]. Another recent study by Pedraza-Sánchez et al. demonstrated significantly better outcomes. Using contrast-enhanced US guidance, they found that 94% of tumors did not require further treatment [6].

One of the factors significantly associated with an increased risk of residual tumor was the final temperature in the ablation zone. According to the manufacturers’ recommendations (Medtronic) at least 65 °C should be aimed for. In 11% of cases, this temperature limit was not achieved, despite an additional ablation period. This was reflected in a higher number of residual tumors discovered at the 3-month CT control (OR 4.23, *p* = 0.006). Several factors have the potential to affect the ablation temperature. Large vessels in the vicinity of the tumors may cause a heat sink that impairs the ability of the RF antenna to produce a temperature high enough to cause tumor necrosis. Endophytic, centrally located tumors are more frequently subjected to this effect than exophytic tumors situated at further distances from larger vessels [30]. Exophytic lesions that are surrounded by perinephric fat will be less prone to heat sinks compared to endophytic tumors [31]. In our study, there was a tendency toward a higher, though not significant, number of endophytic lesions in the group with residual tumors at the first CT follow-up, 62% compared to 55% in the whole group, which is in line with this side effect. In addition, the significantly higher number of residual tumors (OR 2.85, *p* = 0.019) in lesions located ≤4 mm to the renal pelvis (35%), in contrast to those >4 mm from the renal pelvis (15%), is probably also explained by their proximity to larger vessels central in the kidney, resulting in a more pronounced heat sink effect. Similar results have been described previously [13,28].

In our study, a tumor size exceeding 3 cm showed a trend of increasing the risk of residual tumor in the multivariate logistic regression, but it did not reach statistical significance. A similar finding was presented by Acosta Ruiz et al. [13], where tumor size did not appear to be a significant factor affecting primary efficacy, i.e., residual tumor growth.

In several studies, tumor size has been described as an important factor regarding the outcome of RF ablation, as the technique is limited by the size of the ablation zone [24,28,30,31]. If the diameter of the lesion exceeds 3 cm, multiple ablations or cluster electrode techniques have been recommended when using the RF technique [30]. Our results, however, show that a high risk (45%) of residual tumor remains for tumor sizes ≥ 4 cm even with these cluster techniques, warranting extra caution before one decides to treat larger renal tumors with the RF technique.

Interestingly, the technical difficulties reported by the operator were expected to relate to an increased number of patients with residual tumors, but this was not statistically significant (*p* = 0.177). However, in 10 cases, the ablation zone did not include the tumor. Of these cases, three procedures were associated with difficulties in visualizing the tumor and in one procedure there was a dislocation of the kidney when positioning the antenna. This finding supports further the value of contrast-enhanced CT at the end of the procedure to confirm the complete ablation of the tumors [28,29,32,33], especially if the procedure is associated with technical difficulties. Immediate post-ablation control with US is often difficult due to the vaporization of water causing an acoustic shadow in the ablated region [34].

In our study, a high BMI did not seem to be correlated with the outcome in terms of residual tumor occurrence. This was somewhat unexpected, since obesity negatively affects US imaging’s feasibility and quality. However, this finding is supported by Davies et al. [23], who also could not correlate BMI to an impaired outcome after US-guided RFA.

Local tumor progression during the late follow-up (20 months) of the RCC was found to be low in our study, 2%. This can be compared with previous publications showing similar levels of progression and is thus comparable with partial nephrectomy [19,35]. Due to the small number of local tumor progressions (four patients), no meaningful statistical analysis of the potential association with the above-mentioned factors can be made. These results show that residual tumor growth is a more frequent problem than local progression when RCC is treated with US-guided RFA.

The limitations of this study include the retrospective design of the analysis. Since cases with complex anatomy and difficulties in obtaining sufficient imaging quality were treated by means of CT guidance, there is a risk of selection bias in favor of US guidance. In addition, the outcome of the retrospective design, with inclusion extended over several years as patient volumes increased, is subject to a learning curve, as the treating physicians become more used to the technique and selecting patients. Another limitation was the relatively short follow-up time of 20 months, which could increase the risk of missing cases of local tumor progression. Furthermore, a fifth of the biopsies were not representative of the tumor, and only 49% of the representative biopsies had been Fuhrman-graded, which limited the power of our sub-analyses.

## 5. Conclusions

This study has shown that US-guided RFA in small renal masses is associated with a relatively high incidence of residual tumors. Factors associated with worse outcomes in our US-guided ablations study include proximity to the collecting system and failure to achieve the target ablation temperature of 65 °C. Sufficient anatomic delineation to obtain an adequate antenna placement is crucial, especially if the US-guided procedure is associated with technical difficulties. In view of previous studies, CT-guided antenna placement or CT confirmation after US guidance, to ensure adequate positioning, is possibly a safer approach, especially in tumors close to the collecting system.

## Figures and Tables

**Table 1:** Patient demographics.

	Results
Number of patients	159
Median age, years (range)	73 (35–89)
Gender	
Female	54 (34%)
Male	105 (66%)
ASA-score (%)	
I	3 (2%)
II	71 (45%)
III	76 (48%)
IV	9 (5%)

**Table 2:** Tumor characteristics.

	Results
Size, cm, mean ± SD (range)	2.4 ± 0.9 (1.0–5.5)
≥3 cm	51
<3 cm	111
Side	
Right	76
Left	86
Location	
Upper pole	48
Mid renal	59
Lower pole	55
Growth type	
≥50% exophytic	73
<50% exophytic	89
Proximity to calyces/renal pelvis	
≤4 mm	48
>4 mm	114

**Table 3:** Distribution of histopathological findings.

BIOPSY	Number	%	Percent of the Malignant Samples (*n* = 113)
Not representative	33	20	-
Clear cell	57	35.5	51
Papillary	32	20	28
Clear cell papillary	12	7.5	10
Chromophobe	11	7	10
Urothelial cancer	1	1	1
Oncocytoma	14	9	-

**Table 4:** Distribution of ablation temperature at the end of the ablation.

Ablation Temperature	Number of Ablations (*n* = 162)	Percent of Total	Percent of Cases with a Reported Temperature (*n* = 151)
Not reported	11	7	-
≥65 degrees	132	82	88
<65 degrees	19	11	12

**Table 5:** Reported difficulties during the RFA.

Technical Difficulties	Number of Patients
None	111 (69%)
Additional ablation due to signs of remaining vital tumor	2 (1%)
Difficulties in positioning the antenna centrally in the tumor	13 (8%)
Technical problems with the ablation equipment	5 (3%)
Difficulties in visualizing the tumor	27 (17%)
Prolonged procedure due to low temperature	3 (2%)

**Table 6:** Description of early (30 d) complications according to the modified Clavien system.

Complications	Number of Patients (%)
None (Grade 0)	139 (86%)
Presence of complications	23 (14%)
Grade 1	16
Grade 2	6
Grade 3	0
Grade 4	1
Grade 5	0

**Table 7:** Additional treatment of patients diagnosed with residual tumor or local tumor progression during CT follow-up.

Additional Treatment	Residual Tumor (*n* = 34)	Local Tumor Progression (*n* = 4)
Additional ablation	19	3
Surgery	6	1
Surveillance	6	0
Lost during follow-up	3 *	0

* One patient moved to another hospital, one patient died, and the third patient had an oncocytoma.

**Table 8:** Odds ratio (OR) and 95% confidence interval (CI) for the risk of residual tumor at first follow-up after ablation.

	OR	95% CI	*p*-Value
Proximity to calices/renal pelvis < 4 mm	2.85	1.19–6.84	0.019
Ablation temperature < 65 °C	4.23	1.52–11.8	0.006
Size of tumor > 30 mm	2.25	0.95–5.36	0.066
Technical difficulties	1.78	0.77–4.08	0.177

## Data Availability

The datasets generated during the current study are available from the corresponding author upon reasonable request.

## Supplementary Materials

The following supporting information can be downloaded at: https://www.mdpi.com/article/10.3390/curroncol31090392/s1, Table S1: Patient demography, ablation temperature, tumor size and localization in patients with residual tumor/local tumor progression compared with those with complete ablation and no local tumor progression.
