# Supplementary material for: Factors that Affect Outcome of Ultrasound-Guided Radiofrequency Ablation of Renal Masses
Source: Curr Oncol. 2024 Sep 10;31(9):5318–29. doi: 10.3390/curroncol31090392 (PMC11431956; doi:10.3390/curroncol31090392)
Supplement: Supplementary file 1 [file curroncol-31-00392-s001.zip › curroncol-3187467-supplementary.pdf]

Current Oncology

MDPI

Table S1. Patient demography, ablation temperature, tumor size and localization in patients with residual tumor/local tumor progression compared with those with complete ablation and no local tumor progression. .

**Incomplete ablation or local tumor progression (N)**	**Yes (*n* = 38)**	**No (*n* = 121)**
Median Age (Range)	72 (44–89)	73 (35–86)
Male/Female	22/16	83/36
ASA-score (median)	2.7 (1–4)	2.5 (1–4)
Body Mass Index (median)	26.0	26.7
% of patients with ablation temperature > 65° C	73 %	92 %
Proximity to the collecting system < 4 mm	50 %	24 %
% of patients with tumor size > 3 cm	50%	26%
